# The genetic structure of SARS‐CoV‐2 does not rule out a laboratory origin: SARS‐COV‐2 chimeric structure and furin cleavage site might be the result of genetic manipulation

**DOI:** 10.1002/bies.202000240

**Published:** 2020-11-17

**Authors:** Rossana Segreto, Yuri Deigin

**Affiliations:** ^1^ Department of Microbiology University of Innsbruck Innsbruck Austria; ^2^ Youthereum Genetics Inc. Toronto Ontario Canada

**Keywords:** BtCov/4991, furin cleavage site, Gain‐of‐function studies, pangolin CoV, RaTG13, receptor binding domain, SARS‐CoV‐2

## Abstract

Severe acute respiratory syndrome‐coronavirus (SARS‐CoV)‐2′s origin is still controversial. Genomic analyses show SARS‐CoV‐2 likely to be chimeric, most of its sequence closest to bat CoV RaTG13, whereas its receptor binding domain (RBD) is almost identical to that of a pangolin CoV. Chimeric viruses can arise *via* natural recombination or human intervention. The furin cleavage site in the spike protein of SARS‐CoV‐2 confers to the virus the ability to cross species and tissue barriers, but was previously unseen in other SARS‐like CoVs. Might genetic manipulations have been performed in order to evaluate pangolins as possible intermediate hosts for bat‐derived CoVs that were originally unable to bind to human receptors? Both cleavage site and specific RBD could result from site‐directed mutagenesis, a procedure that does not leave a trace. Considering the devastating impact of SARS‐CoV‐2 and importance of preventing future pandemics, researchers have a responsibility to carry out a thorough analysis of all possible SARS‐CoV‐2 origins.

## Graphical abstract

The perfect binding ability of SARS‐CoV‐2 to human cells and the presence of the furin cleavage site, which is new for SARS‐like coronaviruses, might derive from genetic manipulation performed during evolutionary studies. By combining a bat coronavirus backbone and a receptor binding domain from pangolin coronavirus the resulting *chimera* would seem completely natural.

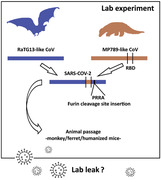

## INTRODUCTION

Nearly a year has passed since the outbreak of severe acute respiratory syndrome‐coronavirus 2 (SARS‐CoV‐2) in Wuhan, China, and its origin is still controversial. Despite the international research effort conducted, a natural host, either direct or intermediate, has not yet been identified. The hypothesis that the Wuhan Huanan Seafood Wholesale Market was the first source for animal–human virus transmission has now been conclusively dismissed^i^ and the few market samples that were collected showed only human‐adapted SARS‐CoV‐2, with no traces of zoonotic predecessor strains^ii^. Almost all scientific papers published to date purport that SARS‐CoV‐2 has a natural origin, and the only published paper considering possible a lab origin^[^ ^1^ ^]^ focuses on serial passage as the technique that could justify SARS‐CoV‐2 special adaptation to human cells. We here describe how the two main SARS‐CoV‐2 features, (1) the presence of a furin cleavage site missing in other CoVs of the same group and (2) an receptor binding domain (RBD) optimized to bind to human cells^[^ ^2^ ^]^ might be the result of lab manipulation techniques such as site‐directed mutagenesis. The acquisition of both unique features by SARS‐CoV‐2 more or less simultaneously is less likely to be natural or caused only by cell/animal serial passage.

## SARS‐COV‐2′S CLOSEST RELATIVES ARE BAT AND PANGOLIN CORONAVIRUSES

Zhou et al.^[^ ^3^ ^]^ from the Wuhan Institute of Virology (WIV) were the first to identify and characterize a new coronavirus (CoV), SARS‐CoV‐2. The genomic sequences obtained from early cases shared 79% sequence identity to the CoVs that caused severe acute respiratory syndrome (SARS‐CoV) in 2002–2003 and 96.2% sequence identity to RaTG13 (MN996532), a CoV sequence detected from a *Rhinolophus affinis* bat. RaTG13 is currently the closest phylogenetic relative for SARS‐CoV‐2 found,^[^ ^4^ ^]^ but its complete genomic sequence was not published before the outbreak of SARS‐CoV‐2 and the original sample was collected in the Yunnan province (China) by the same group of WIV researchers in 2013. Zhou et al.^[^ ^3^ ^]^ stated to have found a match between SARS‐CoV‐2 and a short region of RNA‐dependent RNA polymerase (RdRp) of a CoV in their database and then fully sequenced the original sample collected in 2013, which they called RaTG13.

We discovered that the RdRp of RaTG13 has 100% nucleotide identity with the sequence BtCoV/4991 (KP876546), which was identified by Ge et al.^[^ ^5^ ^]^ in a *Rhinolophus affinis* bat in the Yunnan province in 2013, same location and year as RaTG13. BtCoV/4991 was collected in a mine colonized by bats near Tongguanzhen, Mojiang, Yunnan. The WIV researchers were invited to investigate the mine after six miners there had contracted severe pneumonia in 2012iii, and three of the miners have died.^[^ ^6^ ^]^ The miners have been tasked with clearing out bat droppings in the mine, and the severity of their pneumonia correlated with the duration of exposure to the mine.^[^ ^7^ ^]^ Four miners’ samples subsequently underwent testing at WIV, where Immunoglobulin G (IgG) antibodies against SARS were identified in all samples.^[^ ^8^ ^]^ Considering that only about 5300 people were infected in mainland China during the SARS outbreak of 2002–2004, most of whom resided in Guandong, the odds of four miners in Yunnan retaining antibodies from the 2002–2004 SARS outbreak are negligible. On the other hand, it is possible that the SARS antibody test administered to the miners cross‐reacted with a novel SARS‐like bat virus that the miners had acquired at the mine. Ge et al.^[^ ^5^ ^]^ have identified a number of CoVs in the mine, but based on the phylogenetic analysis, BtCoV/4991 was the only SARS‐related strain, clearly separated from all known alpha‐ and beta‐CoVs at that time. BtCoV/4991 was also different from other bat CoVs in the phylogenetic analysis carried out by Wang et al. in 2019.^[^ ^9^ ^]^ Chen et al.^[^ ^10^ ^]^^ ^identified BtCoV/4991 as the closest sequence to SARS‐CoV‐2 because RaTG13 had not yet been published at that time. BtCoV/4991 and RaTG13 have been later asserted to be two different coding names of the same strain, as their original authors at WIV registered the two strains as one entry in the Database of Bat‐associated Viruses (DBatVir).^iv^

In late July 2020, Zhengli Shi, the leading CoV researcher from WIV, in an email interview ^[^ ^11^ ^]^ asserted the renaming of the RaTG13 sample and unexpectedly declared that the full sequencing of RaTG13 has been carried out as far back as in 2018 and not after the SARS‐CoV‐2 outbreak, as stated in Zhou et al.^[^ ^3^ ^]^ The reversal in WIV's stance on when exactly RaTG13 was fully sequenced could have been due to the discovery by independent researchers into the origins of SARS‐CoV‐2 that the filenames of the raw sequencing reads deposited by WIV on May 19, 2020^v^ seem to indicate that sequencing for RaTG13 was done in 2017 and 2018.^vi^ However, no formal erratum about year of sequencing and sample renaming from the authors of Zhou et al. ^[^ ^3^ ^]^ has yet appeared, or as far as is currently known, has been submitted.

The second non‐human RdRp sequence closest to BtCoV/4991 (91.89% nucleotide identity) is the CoV sequence MP789 (MT084071) isolated in 2019 in a Malaysian pangolin (*Manis javanica*) from the Guangdong province (GD), China.^[^ ^12^ ^]^ The envelope protein of MP789 shows surprisingly 100% aminoacidic identity with the corresponding protein in RaTG13, in bat‐SL‐CoVZXC21 (MG772934.1), in bat‐SL‐CoVZC45 (MG772933.1) and in some early SARS‐CoV‐2 isolates (e.g. YP_009724392).^[^ ^13^ ^]^ The envelope protein of CoVs is involved in critical aspects of the viral lifecycle, such as viral entry, replication and pathogenesis.^[^ ^14^ ^]^

## BAT COVS HAVE BEEN THOROUGHLY STUDIED AND GENETICALLY MANIPULATED

Many studies have reported that bats are natural reservoirs for a broad diversity of potentially pathogenic SARS‐like CoVs.^[^ ^15^, ^16^ ^]^ Some of these viruses can potentially directly infect humans^[^ ^17^ ^]^, whereas others need to mutate their spike protein in order to effectively bind to the human angiotensin 1‐converting enzyme 2 (hACE2) receptor and mediate virus entry.^[^ ^18^ ^]^ In order to evaluate the emergence potential of novel CoVs, researchers have created a number of chimeric CoVs, consisting of bat CoV backbones, normally unable to infect human cells, whose spike proteins were replaced by those from CoVs compatible with human ACE2. These chimeras were meant to simulate recombination events that might occur in nature.^[^ ^19^, ^20^ ^]^ Such gain‐of‐function experiments have raised a number of biosafety concerns and stirred controversy among researchers and the general public. One of the main arguments in favor of gain‐of‐function studies is the need to be prepared with an arsenal of drugs and vaccines for the next pandemic.^[^ ^21^ ^]^ By contrast, one of the main arguments against them is that the next pandemic itself could be caused by those experiments, due to the risk of lab escape.^[^ ^22^, ^23^ ^]^

In recent years, the field of corona‐virology had been focused on pan‐CoV therapies and vaccines, as evident from research conducted in the past 5 years,^[^ ^24^, ^25^, ^26^, ^27^ ^]^ as well as from media reports.vii Synthetically generating diverse panels of potential pre‐emergent CoVs was declared a goal of active grants for the EcoHealth Alliance, which funded some of such research at WIV, in collaboration with laboratories in the USA and other international partners.viii

## CREATING CHIMERIC COVS WITH NOVEL RBDS HAS GONE ON FOR DECADES

Researchers have been generating chimeric CoVs for over two decades, long before the advent of modern sequencing or genetic engineering techniques. For example, in 1999, a group from Utrecht University used targeted RNA recombination to create a “cat‐and‐mouse” CoV chimera: the RBDs of a feline and murine CoV were swapped, demonstrating that this exchange swapped also species tropism during *in vitro* experiments.^[^ ^28^ ^]^

In 2007, the Shi group at WIV created a series of “bat‐man” CoV chimeric spike proteins while trying to determine what exactly confers CoVs the ability to jump from one species to another. The researchers used different segments of the spike protein of the human SARS virus to replace corresponding segments in the spike protein of a bat viral backbone. It was concluded that a relatively short region (aa 310 to 518) of the spike protein “was necessary and sufficient to convert Rp3‐S into a huACE2‐binding molecule,”^29^ that is to provide the bat CoV spike protein with a novel ability of binding to a human ACE2 receptor.

In 2008, the Baric group at the University of North Carolina (UNC) took the WIV research one step further: instead of using human immunodeficiency viruses (HIV) pseudo‐viruses with bat CoV spike proteins, a live chimeric CoV was created. Following the experiments of their 2007 WIV colleagues, the Baric group used a bat SARS‐like CoV as a backbone and replaced its RBD with the RBD from human SARS.^[^ ^30^ ^]^

In 2015, the Shi and Baric groups joined forces and published probably the most famous gain‐of‐function virology paper, which described the creation of another synthetic chimeric virus.^[^ ^19^ ^]^ This time the RBD of a mouse‐adapted SARS backbone (SARS‐MA15) was replaced by the RBD of RsSHC014, a bat strain previously isolated from Yunnan bats in 2011 by the Shi group. In 2016, the Baric group repeated their 2015 experiment using the same SARS‐MA15 backbone and the RBD from Rs3367,^[^ ^31^ ^]^ a close relative of RsSHC014 also previously found in Yunnan by WIV and renamed “WIV1” after live culturing.^[^ ^17^ ^]^

Probably the largest reported number of novel chimeric viruses created was described in a 2017 paper from the Shi group at WIV,^[^ ^15^ ^]^ in which the authors reported creating eight chimeric viruses using WIV1 as a backbone and transplanting into it various RBDs from bat SARS‐like viruses. These viruses were collected over a span of 5 years from the same cave near Kunming, Yunnan Province, where the Shi group originally found Rs3367 and RsSHC014. Only two of the eight live chimeric viruses were successfully rescued, and those two strains were found to possess the ability to bind to the human ACE2 receptor, as confirmed by experiments in hACE2‐expressing HeLa cells and RT‐PCR quantification of viral RNA.

## SARS‐COV‐2 SHARES ITS RBD WITH A PANGOLIN COV

The possibility that pangolins could be the intermediate host for SARS‐CoV‐2 has long been under discussion. ^[^ ^32^, ^33^, ^34^ ^]^ The biggest divergence between SARS‐CoV‐2 and RaTG13 is observed in the RBD of their spike proteins.^[^ ^4^ ^]^ Although its overall genome similarity is lower to SARS‐CoV‐2 than that of RaTG13, the MP789 pangolin strain isolated from GD pangolins has an almost identical RBD to that of SARS‐CoV‐2. Indeed, pangolin CoVs and SARS‐CoV‐2 possess identical amino acids at the five critical residues of the RBD, whereas RaTG13 only shares one amino acid with SARS‐CoV‐2.^[^ ^35^ ^]^ ACE2 sequence similarity is higher between humans and pangolins than between humans and bats. Intriguingly, the spike protein of SARS‐CoV‐2 has a higher predicted binding affinity to human ACE2 receptor than to that of pangolins and bats.^ix^ Before the SARS‐CoV‐2 outbreak, pangolins were the only mammals other than bats documented to carry and be infected by SARS‐CoV‐2 related CoV.^[^ ^12^ ^]^ Recombination events between the RBD of CoV from pangolins and RaTG13‐like backbone could have produced SARS‐CoV‐2 as chimeric strain. For such recombination to occur naturally, the two viruses must have infected the same cell in the same organism simultaneously, a rather improbable event considering the low population density of pangolins and the scarce presence of CoVs in their natural populations.^x^ Moreover, receptor binding studies of reconstituted RaTG13 showed that it does not bind to pangolin ACE2.^xi^

## THE FURIN CLEAVAGE SITE: THE KEY DIFFERENCE BETWEEN SARS‐COV‐2 AND ITS CLOSEST RELATIVE RATG13

SARS‐CoV‐2 differs from its closest relative RaTG13 by a few key characteristics. The most striking difference is the acquisition in the spike protein of SARS‐CoV‐2 of a cleavage site activated by a host‐cell enzyme furin, previously not identified in other beta‐CoVs of lineage b^[^ ^36^ ^]^ and similar to that of Middle East respiratory syndrome (MERS) coronavirus.^[^ ^35^ ^]^ Host protease processing plays a pivotal role as a species and tissue barrier and engineering of the cleavage sites of CoV spike proteins modifies virus tropism and virulence.^[^ ^37^ ^]^ The ubiquitous expression of furin in different organs and tissues have conferred to SARS‐CoV‐2 the ability to infect organs usually invulnerable to other CoVs, leading to systemic infection in the body.^[^ ^38^ ^]^ Cell‐cultured SARS‐CoV‐2 that was missing the above‐mentioned cleavage site caused attenuated symptoms in infected hamsters,^[^ ^39^ ^]^ and mutagenesis studies have confirmed that the polybasic furin site is essential for SARS‐CoV‐2′s ability to infect human lung cells.^[^ ^40^ ^]^

**FIGURE 1:**
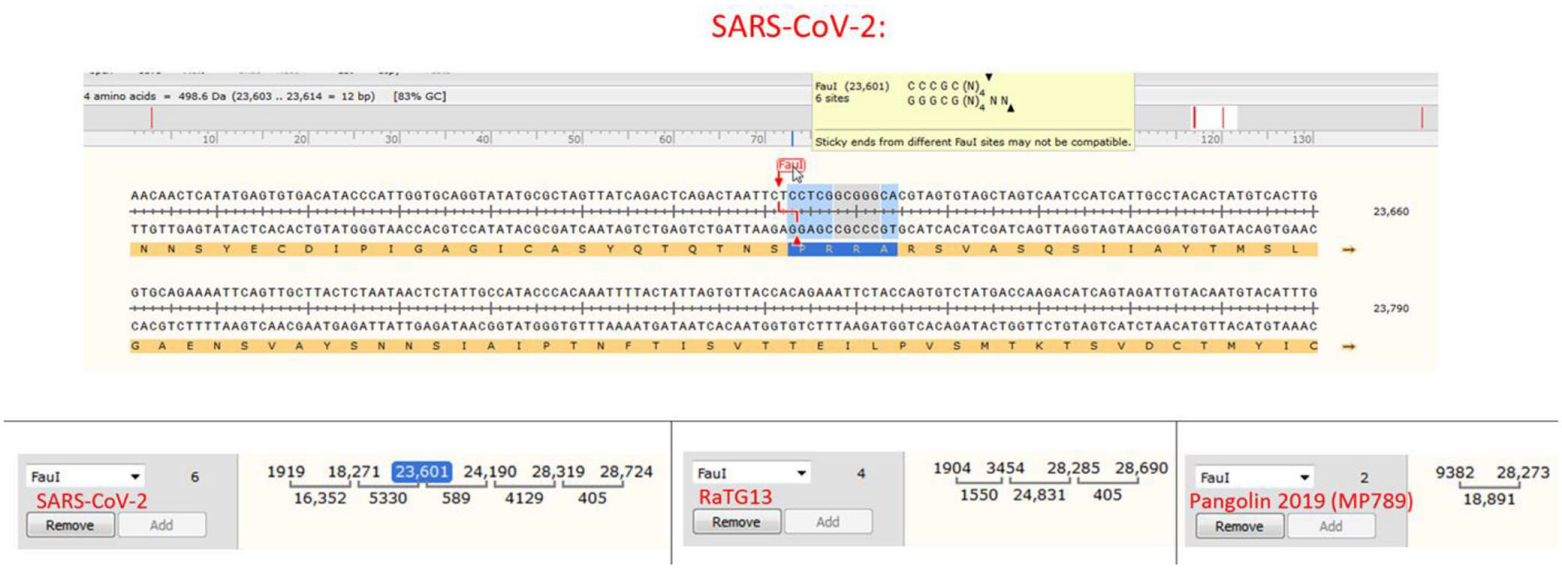
Nucleotide sequence of the S protein at the S1/S2 junction in SARS‐CoV‐2 (NC045512.2) showing the furin cleavage site (in blue) that includes a *FauI* enzyme restriction site

The polybasic furin site in SARS‐CoV‐2 was created by a 12‐nucleotide insert TCCTCGGCGGGC coding for a PRRA amino acid sequence at the S1/S2 junction (Figure 1). Interestingly, the two joint arginines are coded by two CGGCGG codons, which are rare for these viruses: only 5% of arginines are coded by CGG in SARS‐CoV‐2 or RaTG13, and CGGCGG in the new insert is the only doubled instance of this codon in SARS‐CoV‐2. The CGGCGG insert includes a *Fau*I restriction site, of which there are six instances in SARS‐CoV‐2 and four instances in RaTG13 (and two in MP789). The serendipitous location of the *Fau*I site could allow using restriction fragment length polymorphism (RFLP) techniques ^[^ ^41^ ^]^ for cloning ^[^ ^42^ ^]^ or screening for mutations, ^[^ ^43^ ^]^ as the new furin site is prone to deletions *in vitro*.^[^ ^39^, ^44^ ^]^

**FIGURE 2:**
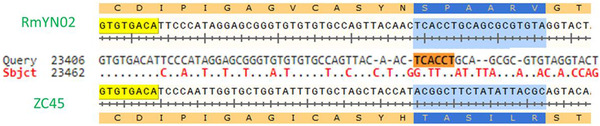
Alignment of nucleotide and amino acid sequences of the S protein from bat‐SL‐CoVZC45 (MG772933.1) and RmYN02 at the S1/S2 junction site. No insertions of nucleotides possibly evolving in a furin cleavage site can be observed (in blue)

A study by Zhou et al.^[^ ^45^ ^] ^reported the discovery of a novel CoV strain RmYN02, which the authors claim exhibits natural PAA amino acid insertions at the S1/S2 cleavage site where SARS‐CoV‐2 has the PRRA insertion. However, upon close examination of the underlying nucleotide sequence of RmYN02 in comparison with its closest ancestors bat‐SL‐CoVZC45 and bat‐SL‐CoVZXC21, no insertions are apparent, just nucleotide mutations (Figure 2).

**FIGURE 3:**
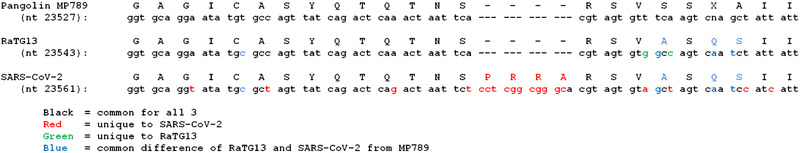
Alignment of nucleotide and amino acid sequences of the S protein from RaTG13 (MN996532), MP789 (MT084071) and SARS‐CoV‐2 (NC045512.2) at the S1/S2 site. The common nucleotides and amino acids are given in black, SARS‐CoV‐2 unique nucleotides and amino acids in red, RaTG13 unique nucleotides and amino acids in green and common nucleotides and amino acids in SARS‐CoV‐2 and RaTG13 that differ in MP789 in blue. The codon forserine (TCA) in RaTG13 and MP789 is split in SARS‐CoV‐2 to give part of a new codon forserine (TCT) and part of the amino acidalanine (GCA)

Therefore, SARS‐CoV‐2 remains unique among its beta CoV relatives not only due to a polybasic furin site at the S1/S2 junction, but also due to the four amino acid insert PRRA that had created it. The insertion causes a split in the original codon for serine (TCA) in MP789 or RaTG13 to give part of a new codon for serine (TCT) and part of the amino acid alanine (GCA) in SARS‐CoV‐2 (Figure 3).

The insertion of the furin cleavage site in SARS‐CoV‐2 is not in frame with the rest of the sequence, when compared with the MP789 and the RaTG13 sequences (Figure 3). Therefore, it is possible to exclude that such insertion could have originated by polymerase slippage or by releasing and repriming, because insertion mutations generated by these mechanisms have been postulated to maintain the reading frame of the viral sequence.^[^ ^46^ ^]^ The possibility that the furin cleavage site could have been acquired by recombination has been recently questioned by Seyran et al.,^[^ ^47^ ^]^ because the SARS‐CoV‐2 spike protein seems to lack any further recombination event in contrast with the recombination model of other CoVs.

## CRITIQUE OF “THE PROXIMAL ORIGIN OF SARS‐COV‐2″

Due to the broad‐spectrum of research conducted over almost 20 years on bat SARS‐CoVs justified by their potential to spill over from animal to human,^[^ ^48^ ^]^ a possible synthetic origin by laboratory engineering of SARS‐CoV‐2 cannot be excluded. The widely cited article of Andersen et al.^[^ ^2^ ^] ^stated that SARS‐CoV‐2 has most likely a natural origin. The main argument brought by the authors is that the high‐affinity binding of the SARS‐CoV‐2 spike protein to hACE2 could not have been predicted by models based on the RBD of SARS‐CoV. Based on the structural analysis conducted by Wan et al.,^[^ ^49^ ^] ^SARS‐CoV‐2 has the potential to recognize hACE2 more efficiently than the SARS‐CoV, which emerged in 2002. Moreover, generation of CoV chimeric strains has recently demonstrated that bat CoV spikes can bind to the hACE2 receptor with more plasticity than previously predicted.^[^ ^15^ ^]^ All amino acids in the RBD have been extensively analyzed and new models to predict ACE2 affinity are available.^[^ ^50^ ^]^ In this regard, BatCoV Rs3367 (99.9% identity to WIV1) has been shown to share with SARS‐CoV‐2 four out of six critical residues in the RBD. Considering that WIV1 was shown to directly bind to hACE2, the same assumption could easily have been made about SARS‐CoV‐2 RBD.^[^ ^51^ ^]^

As described above, creation of chimeric viruses has been carried out over the years with the purpose of studying the potential pathogenicity of bat CoVs for humans. In this context, SARS‐CoV‐2 could have been synthesized by combining a backbone similar to RaTG13 with the RBD of CoV similar to the one recently isolated from pangolins^[^ ^12^ ^]^, because the latter is characterized by a higher affinity with the hACE2 receptor. Such research could have aimed to identify pangolins as possible intermediate hosts for bat‐CoV potentially pathogenic for humans. Subsequent serial cell or animal passage, as described by Sirotkin & Sirotkin ^[^ ^1^ ^]^ could have provided the perfect adaptation of the RBD to the hACE2.

Regarding the furin cleavage site, Andersen et al.^[^ ^2^ ^]^ state that “the functional consequence of the polybasic cleavage site in SARS‐CoV‐2 is unknown.” New studies from several groups have lately identified this activation site as possibly enabling the virus to spread efficiently between humans and attack multiple organs.^[^ ^52^ ^]^ Experiments on proteolytic cleavage of CoV spike proteins have been recently suggested as future key studies to understand virus transmissibility in different hosts.^[^ ^50^ ^]^

Andersen et al.^[^ ^2^ ^]^ also state, based on the work of Almazan et al.^[^ ^53^ ^]^ that “the genetic data irrefutably show that SARS‐CoV‐2 is not derived from any previously used virus backbone.” In the last 6 years before the outbreak of SARS‐CoV‐2 the number of potential bat backbones has been undeniably increased by several bat CoV screenings, last but not least bringing RaTG13 to scientific attention in January 2020. Other possible backbones could, as well, still wait for publication.

Andersen et al.^[^ ^2^ ^]^ affirm that “the acquisition of both the polybasic cleavage site and predicted O‐linked glycans also argues against culture‐based scenarios.” Methods for insertion of a polybasic cleavage site in infectious bronchitis CoV are given in Cheng et al.^[^ ^54^ ^]^ and resulted in increased pathogenicity. Concerning the predicted O‐linked glycans around the newly inserted polybasic site, it should be noted that this prediction was not confirmed by Cryo‐EM inquiry into the SARS‐CoV‐2 spike glycoprotein.^[^ ^55^ ^]^ Nevertheless, while it is true that O‐linked glycans are much more likely to arise under immune selection, they could be added in the lab through site‐directed mutagenesis^[^ ^56^ ^]^ or arise in the course of *in vivo* experiments, for example, in BLT‐L mice with human lung implants and autologous human immune system^[^ ^57^ ^]^ or in mice expressing the hACE2 receptor.^[^ ^31^ ^]^ To overcome problems of bat CoV isolation, experiments based on direct inoculation of bat CoV in suckling rats have been carried out.^[^ ^58^ ^]^ Humanized mice, ferrets, primates and/or other animals with similar ACE2 conformation could have all been used for serial passage experiments, as described in detail by Sirotkin and Sirotkin.^[^ ^1^ ^]^

Andersen et al.^[^ ^2^ ^]^ also state that “subsequent generation of a polybasic cleavage site would have then required repeated passage in cell culture or animals with ACE2 receptors similar to those of humans, but such work has also not previously been described.” It should not be excluded that such experiments could have been aborted due to the SARS‐CoV‐2 outbreak, before a possible publication of the results or that the results were never intended to be published.

It is important to mention that RaTG13 and the pangolin CoV sequences from smuggled pangolins confiscated in the GD province in March 2019, and to which most of published papers supporting a natural origin of SARS‐CoV‐2 refer,^[^ ^2^ ^]^ have recently been questioned as to the accuracy of their assembly dataxii and require further analyses to prove their correctness.^[^ xiii ^,^ xiv ^]^ It should also be noted that *in vitro* receptor binding studies of reconstituted RaTG13 yielded some peculiar results.^[xi]^ The most surprising observation was that RaTG13, unlike SARS‐CoV‐2, is unable to bind ACE2 in *R. macrotis* bats, a close relative of RaTG13's purported host, *R. affinis* ^[^ ^59^ ^]^ (whose ACE2 receptor has not yet been tested). At the same time, RaTG13 was observed to bind hACE2^[^ ^60^ ^]^, but not as well as ACE2 of rats and mice, to which SARS‐CoV‐2 did not bind at all. Is it possible that just as SARS‐MA15 was a mouse‐adapted strain of SARS, RaTG13 is actually a mouse‐adapted version of a CoV extracted from the Mojiang cave, rather than a strain obtained from a bat fecal swab? Unfortunately, the RaTG13 sample has been exhausted and it is no longer available for external examination,^[^ ^11^ ^]^ which is unfortunate given a number of inconsistencies in its sequencing raw data. Also, the status and availability of the Mojiang miners’ samples remain as well an open and highly relevant question. Several samples from the miners have been collected^[^ ^7^, ^8^ ^]^ and likely stored, and it would be of great value to test them for the presence of SARS‐CoV‐2‐like CoVs.

Another open question is the reason for modification and subsequent deletion of WIV's own viral database. In May 2020, several media outlets have reported that the change tracking system of WIV's internal database showed that the database was renamed from “Wildlife‐borne viral pathogen database” to “Bat and rodent‐borne viral pathogen database,” and its description was edited to replace instances of “wild animal” by “bat and rodent”; in addition, mention of “arthropod vectors” was deleted.^xv^ The database description reported that it contained over 60 Mb of data in structured query language (SQL) format, but at as of early May 2020 the download link no longer worked.xvi Subsequently, the database page was taken down in its entirety but its snapshot is still available on Web Archive.xvii It is possible that other international CoV labs might have downloaded the SQL archive of the WIV database before it was taken down, in which case such groups should make those data publicly available.

## HOW COULD THE VIRUS HAVE ESCAPED FROM A LAB?

The leak of highly dangerous pathogens from laboratories is not a rare event and occurrences have been documented in several countries. The most notable lab leak known is the 1977 H1N1 lab escape from China that caused a worldwide pandemic.^[^ ^61^ ^]^ The most recent one is the November 2019 outbreak of brucellosis that occurred in two research centers in Lanzhou, China, infecting over 100 students and staff members.^[^ ^62^ ^]^ Several lab escapes of the first SARS virus have been reported as well: in the summer of 2003 in Singapore,^[^ ^63^ ^]^ then in December 2003 in Taiwan,xviii and in the spring of 2004 twice in China.xix

Concerns about WIV's lab safety were raised in 2018 by U.S. Embassy officials after visiting the Institute and having an interview with Zhengli Shi. The lab auditors summarized their worries in subsequent diplomatic cables to Washington.^xx^ Chinese experts have also raised concerns about lab safety in their own country, lamenting that “lab trash can contain man‐made viruses, bacteria or microbes” and that “some researchers discharge laboratory materials into the sewer after experiments without a specific biological disposal mechanism.”xxi

American labs have also had their share of safety issues. Recently, research operations in the Biosafety level (BSL)‐4 United States Army Medical Research Institute of Infectious Diseases (USAMRIID) facility in Fort Detrick were interrupted in August 2019 following safety violations, in particular, relating to the disposal of infective materials.xxii Other US labs have been cited for safety issues as well.^22^ ^]^

A number of scenarios causing SARS‐CoV‐2 to leak from a lab can be hypothesized. For example, an infected animal could have escaped from a lab or it could have scratched or bitten a worker (a concern raised in 2017 about the establishment of a BSL‐4 primate vaccine testing facility in Kunming, Yunnan^[^ ^64^ ^]^), or a researcher could have accidentally stuck themselves with inoculate (as happened in two cases in Russiaxxiii). Until 2020, CoVs were not considered particularly deadly or virulent. SARS‐like CoVs did not require BSL‐4 and could be manipulated under BSL‐2 and BSL‐3^[^ ^42^ ^]^ conditions, making an accidental leak more likely. Aerosol experiments with CoVs^[^ ^65^ ^]^ could result in lab leak as well, because a failure in the equipment used could go unnoticed for a long time before infection of lab workers is detected. Finally, the virus could potentially have leaked through the sewage system if proper waste disposal and/or decontamination procedures were not followed.

## CONCLUSIONS AND OUTLOOK

On the basis of our analysis, an artificial origin of SARS‐CoV‐2 is not a baseless conspiracy theory that is to be condemned^[^ ^66^ ^]^ and researchers have the responsibility to consider all possible causes for SARS‐CoV‐2 emergence. The insertion of human‐adapted pangolin CoV RBD obtained by cell/animal serial passage and furin cleavage site could arise from site‐directed mutagenesis experiments, in a context of evolutionary studies or development of pan‐CoV vaccines or drugs. A recent article in Nature^[^ ^67^ ^]^ affirms that a laboratory origin for SARS‐CoV‐2 cannot be ruled out, as researchers could have been infected accidentally, and that gain‐of‐function experiments resulting in SARS‐CoV‐2 could have been performed at WIV. Genetic manipulation of SARS‐CoV‐2 may have been carried out in any laboratory in the world with access to the backbone sequence and the necessary equipment and it would not leave any trace. Modern technologies based on synthetic genetics platforms allow the reconstruction of viruses based on their genomic sequence, without the need of a natural isolate.^[^ ^68^ ^]^

A thorough investigation on strain collections and research records in all laboratories involved in CoV research before SARS‐CoV‐2 outbreak is urgently needed. Special attention should be paid to strains of CoVs that were generated in virology laboratories but have not yet been published, as those possibly described in the deleted WIV database. Because finding a possible natural host could take years, as with the first SARS,^[^ ^67^ ^]^ or never succeed, equal priority should be given to investigating natural and laboratory origins of SARS‐CoV‐2.

Xiao Qiang, a research scientist at Berkeley, recently stated: “To understand exactly how this virus has originated is critical knowledge for preventing this from happening in the future.”^[xxi]^

## CONFLICT OF INTEREST

Rossana Segreto and Yuri Deigin do not have any conflicts of interest.

## Data Availability

Data sharing not applicable to this article as no datasets were generated or analysed during the current study.

## Footnotes

i Areddy, J. T. (**2020**). China rules out animal market and lab as coronavirus origin. *The* *Wall Street Journal*. https://www.wsj.com/articles/china-rules-out-animal-market-and-lab-as-coronavirus-origin-11590517508 (last accessed on Oct 15, 2020).

ii Zhan, S. H., Deverman, B. E., Chan, Y. A. (**2020**). SARS‐CoV‐2 is well adapted for humans. What does this mean for re‐emergence? *BioRxiv*. https://doi.org/10.1101/2020.05.01.073262 (last accessed on Oct 15, 2020).

iii Qiu, J. (**2020**). How China's ‘Bat Woman’ hunted down viruses from SARS to the new coronavirus. *Sci. Am*. https://www.scientificamerican.com/article/how-chinas-bat-woman-hunted-down-viruses-from-sars-to-the-new-coronavirus1/ (last accessed on Oct 15, 2020).

iv DBatVir – *The Database of Bat‐Associated Viruses*. http://www.mgc.ac.cn/cgi-bin/DBatVir/main.cgi?func=accession&acc=MN996532 (last accessed on Oct 15, 2020).

v SRX8357956: amplicon sequences of RaTG13. https://www.ncbi.nlm.nih.gov/sra/SRX8357956 (last accessed on Oct 15, 2020).

vi Anon. (**2020**). Names of the RaTG13 amplicon sequences. https://web.archive.org/web/20200918174030/https://graph.org/RaTG13‐Amplicon‐Names‐07‐03 (last accessed on Oct 15, 2020).

vii Kahn, J. (**2020**). How scientists could stop the next pandemic before it starts. *NYT* Magazine. https://www.nytimes.com/2020/04/21/magazine/pandemic-vaccine.html (last accessed on Oct 15, 2020).

viii Project Number 2R01AI110964‐06, *ECOHEALTH ALLIANCE, INC*., https://projectreporter.nih.gov/project_info_description.cfm?aid=9819304&icde=49645421&ddparam=&ddvalue=&ddsub=&cr=1&csb=default&cs=ASC&pball= (last accessed on Oct 15, 2020).

ix Piplani, S., Singh, P. K., Winkler, D. A., Petrovsky, N. (**2020**). In silico comparison of spike protein‐ACE2 binding affinities across species; significance for the possible origin of the SARS‐CoV‐2 virus.*arXiv*. http://arxiv.org/abs/2005.06199 (last accessed on Oct 15, 2020).

x Lee, J., Hughes, T., Lee, M.‐H., Field, H., Rovie‐Ryan, J. J., Sitam, F. T., … Daszak, P. (**2020**). No evidence of coronaviruses or other potentially zoonotic viruses in Sunda pangolins (*Manis javanica*) entering the wildlife trade *via* Malaysia. *BioRxiv*. https://doi.org/10.1101/2020.06.19.158717 (last accessed on Oct 15, 2020).

xi Mou, H., Quinlan, B. D., Peng, H., Guo, Y., Peng, S., Zhang, L., … Farzan, M. (**2020**). Mutations from bat ACE2 orthologs markedly enhance ACE2‐Fc neutralization of SARS‐CoV‐2. *BioRxiv*. https://doi.org/10.1101/2020.06.29.178459 (last accessed on Oct 15, 2020).

xii Zhang, D. (2020). Anomalies in BatCoV/RaTG13 sequencing and provenance. *Zenodo*. https://zenodo.org/record/3969272 (last accessed on Oct 15, 2020).

xiii Singla, M., Ahmad, S., Gupta, C., Sethi, T. (**2020**). De novo assembly of RaTG13 genome reveals inconsistencies further obscuring SARS‐CoV‐2 origins. *Preprints*. https://doi.org/10.20944/preprints202008.0595.v1 (last accessed on Oct 12, 2020).

xiv Chan, Y. A., Zhan, S. H. (**2020**). Single source of pangolin CoVs with a near identical spike RBD to SARS‐CoV‐2. *BioRxiv*. https://doi.org/10.1101/2020.07.07.184374 (last accessed on Oct 15, 2020).

xv Devine, M. (**2020**). What is China covering up about the coronavirus? *NYT* Magazine. https://nypost.com/2020/05/06/what-is-china-covering-up-about-the-coronavirus-devine/ (last accessed on Oct 12, 2020).

xvi
https://twitter.com/ydeigin/status/1259891518468427776 (last accessed on Oct 15, 2020).

xvii Bat and rodent‐borne viral pathogen database. https://web.archive.org/web/20200529174243/http://csdata.org/p/308/ (last accessed on Oct 15, 2020).

**xviii:** Reuters (**2003**). SARS case confirmed in Taiwan. *Wired*. https://www.wired.com/2003/12/sars-case-confirmed-in-taiwan/ (last accessed on Oct 13, 2020).

xix Walgate, R. (**2004**). SARS escaped Beijing lab twice. *The Scientist Magazine*. https://www.the-scientist.com/news-analysis/sars-escaped-beijing-lab-twice-50137 (last accessed on Oct 15, 2020).

xx Rogin, J. (**2020**). State Department cables warned of safety issues at Wuhan lab studying bat coronaviruses. *The Washington Post*. https://www.washingtonpost.com/opinions/2020/04/14/state-department-cables-warned-safety-issues-wuhan-lab-studying-bat-coronaviruses/ (last accessed on Oct 15, 2020).

xxi Caiyu, L., Shumei, L. (**2020**). Biosafety guideline issued to fix chronic management loopholes at virus labs. *Global Times*. https://www.globaltimes.cn/content/1179747.shtml (last accessed on Oct 15, 2020).

xxii Grady, D. (**2020**). Deadly germ research is shut down at army lab over safety concerns. *NYT* Magazine. https://www.nytimes.com/2019/08/05/health/germs-fort-detrick-biohazard.html (last accessed on Oct 15, 2020).

**xxiii:** Miller, J. (**2004**). Russian scientist dies in Ebola accident at former weapons Lab. *NYT* Magazine. https://www.nytimes.com/2004/05/25/world/russian-scientist-dies-in-ebola-accident-at-former-weapons-lab.html (last accessed on Oct 15, 2020).
